# Antioxidant and alpha‐amylase inhibitory potentials of *Cocos nucifera* husk

**DOI:** 10.1002/fsn3.741

**Published:** 2018-07-20

**Authors:** Hamdalat Folake Muritala, Jubril Olayinka Akolade, Sarah Abimbola Akande, Azeemat Titilola Abdulazeez, Raliat Abimbola Aladodo, Abdulkabir Bolakale Bello

**Affiliations:** ^1^ Department of Biochemistry University of Ilorin Ilorin Nigeria; ^2^ Biotechnology Advanced Research Centre Sheda Science and Technology Complex Abuja Nigeria; ^3^ Department of Biological Science Al‐Hikmah University Ilorin Nigeria; ^4^ Department of Biochemistry Kwara State Univesity Malete Nigeria

**Keywords:** alpha‐amylase, antioxidants, *Cocus nucifera*, diabetes mellitus, functional foods

## Abstract

Concoctions containing extract from *Cocos nucifera* husk fiber are used in Nigeria by traditional medicine practitioners for management of diabetes and its associated complications. Preliminary antidiabetic study was designed to validate the folkloric usage of the plant extract. Dried coconut husk fiber was pulverized and extracted with methanol, followed by partitioning of the methanolic extract in ethyl acetate. Phenolic content, radical scavenging activity and antioxidant capacity as well as inhibitory effects of *C. nucifera* methanolic (CN‐M) extract and its ethyl acetate (CN‐E) fraction on pancreatic α‐amylase and lipid peroxidation were determined. Total phenolic content and antioxidant capacity of CN‐E fraction were significantly higher than that of CN‐M extract, whereas there was no significant difference in their ability to scavenge free radicals. The CN‐E fraction also exhibited higher in vitro and in vivo inhibitory effects on α‐amylase activity and lipid peroxidation; reducing blood glucose level within 5 days following intraperitoneal administration of the *C. nucifera* extract to alloxan‐induced hyperglycemic rats. The phenolic‐rich extracts from coconut husk can be further explored as nutraceutical supplement in food formulation for diabetic patients.

## 1 INTRODUCTION

Traditional medicine employs the use of natural products for the management of noncommunicable diseases such as diabetes, cardiovascular and inflammatory diseases, due to the high cost of available orthodox therapy and adverse effect of synthetic drugs. Herbal preparations (concoctions, decoctions and infusions) are prescribed to patients for the management of diabetes and its associated secondary complications. In north central Nigeria, personal communication with traditional medicine practitioners (TMPs) revealed that diabetic diets are formulated with extracts from the dried husk of *Cocos nucifera* (coconut).

*Cocos nucifera* (Linn) belongs to the family Arecaceae; widely grown in tropic and subtropic regions, and are of great economic importance. Traditionally in Nigeria, *C. nucifera* has been used for treatment of hypertension and diabetes (Gbolade, 2012; Soladoye, Chukwuma, & Owa, 2012). Research studies have also reported analgesic and anti‐inflammatory activities (Dua et al., 2013), antimicrobial and antiviral (Esquenazi et al., 2002). Antidiabetic properties of different part of the plant such as the water, oil, and fruit juice have been reported in literature (Mohammed & Luka, 2013; Naskar et al., 2011).

Diabetes mellitus is a group of metabolic disorders characterized by hyperglycemia associated with absolute or relative deficiencies in insulin secretion or insulin action resulting in alteration of carbohydrate, fat, and protein metabolism (American Diabetes Association, 2014). An imbalance in the body's defense mechanism (antioxidants) and free radicals produced leads to oxidative stress which plays a very important role in the pathogenesis of the disease (Maritim, Sanders, & Watkins, 2003). A beneficial therapeutic approach for the treatment of diabetes is to lower postprandial hyperglycemia by inhibiting carbohydrate hydrolyzing enzymes, this will delay the degradation of complex carbohydrate, increase carbohydrate digestion time, and slow down the absorption of glucose (Salehi, Asghari, Esmaeili, Dehghan, & Ghazi, 2013). Several management options for diabetes such as insulin releasers, insulin sensitizers, sulfonylureas and biagunides, α‐amylase and α‐glucosidase inhibitors are associated with serious shortcomings like limited and decreased efficacy over time and ineffectiveness against some long‐term diabetic complications. Acarbose, voglibose, and migilitol are examples of alpha‐amylase inhibitors used in management of diabetes mellitus, they are synthetic compounds associated with various side effects such as flatulence, diarrhea, bloating, and abdominal pain, thus, the need to explore other natural/nonsynthetic alternatives with fewer side effects (Bhutkar & Bhise, 2012; Salehi et al., 2013).

Plants are storehouse of traditional and modern medicine; medicinal and pharmaceutical properties of plants are associated with chemical substances such as polyphenols they produce and store as secondary metabolites. In addition to glucose lowering properties, bioactives from plants have been show to play important role in α‐glucosidase and α‐amylase inhibition, scavenging of free radicals, and decreasing the rate of lipid peroxidation (Hanhineva et al., 2010). Various plant parts of *C. nucifera* has been used for management of diabetes, however, there is little knowledge on the use of the dried husk, hence, the aim of this study was to investigate the in vitro and in vivo antioxidant and alpha‐amylase inhibitory potentials of polyphenolic extracts of the dried husk of *C. nucifera*.

## 2 MATERIALS AND METHODS

### 2.1 Collection of plant sample

Matured coconut fruit with dried brownish husk was collected from a single population within the premises of Adebayo Estate Tanke, Ilorin, Nigeria. The plant sample was authenticated at the Herbarium unit of the Department of Plant Biology, University of Ilorin, Ilorin, Nigeria.

### 2.2 Chemicals and reagents

Folin‐Ciocalteu reagent, 1,1‐diphenyl‐2‐picraylhydrazyl (DPPH), and pancreatic α‐amylase were products from Sigma‐Aldrich (St. Louis, Mo, USA); 3,5‐dinitrosalicylic acid (DNS), trichloroacetic acid, thiobarbituric acid, sulfuric acid, sodium phosphate, and ammonium molybdate, sodium nitropruside, sodium dodeacyl sulfate were products of BDH Laboratories (Poole, UK), while methanol and ethyl acetate were from JHD (China). All reagents were supplied locally by Shepha Biotech Ventures (Lagos, Nigeria). All other chemicals used were of research and analytical grade.

### 2.3 Experimental animals

Rats weighing between 128 and 152 g were obtained from the Animal Holding Unit of the Department of Biochemistry, University of Ilorin, Ilorin, Nigeria. The rats were acclimatized for 2 weeks to standard housing condition, water, and feed pellets (Top Feeds Limited, Lagos, Nigeria) were provided ad‐libitum. The research adheres strictly and conforms to the Principles of Laboratory Animal Care (NIH Publication No. 85‐23). The research protocols were requested to and approved by the Department of Biochemistry, University of Ilorin, Ilorin, Nigeria.

### 2.4 Preparation of plant extracts

**Figure 1:**
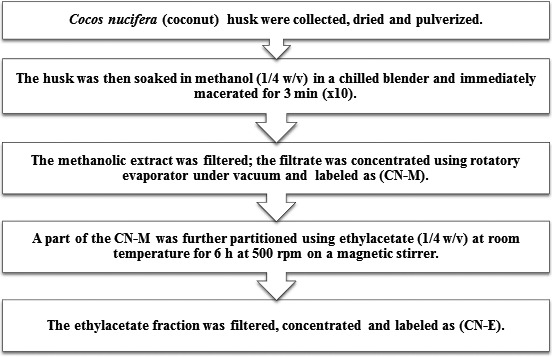
Schematic representation for preparation of Cocos nucifera methanolic extract (CN‐M) and its ethyl acetate fraction (CN‐E)

The dried brownish husk was separated from the *C. nucifera* fruits, further air‐dried for 2 weeks, and then pulverized. Extraction was carried out with methanol, followed by partitioning of the methanol extract in ethyl acetate as shown in Figure 1.

### 2.5 Determination of phenolic content

Total polyphenolic content (TPC) of CN‐M extract and CN‐E fraction were determined using Folin‐Ciocalteu reagent (Singleton, Orthofer, & Lamuela‐Raventós, 1999) with slight modifications. Stock solutions of CN‐M and CN‐E (10 mg/ml) were prepared and further diluted to give 1 mg/ml. The diluted solutions (0.1 ml) were mixed with 0.2 N Folin‐Ciocalteu reagent (1 ml) for 5 min before 0.9 ml of 1 M Na_2_CO_3_ solution was added. The mixtures were allowed to stand for 30 min and the absorbance was determined at 765 nm. Standard curve of gallic acid (0.005–0.1 mg/ml) used as reference phenolic compound was also prepared. TPC of extracts were derived by regressing absorbance readings of test samples onto the standard curve and expressed as mg gallic acid equivalent per gram of sample.

### 2.6 Determination of radical scavenging activity

DPPH radical scavenging activity of CN‐M extract and CN‐E fraction were determined (Alam, Bristi, & Rafiquzzaman, 2013) with slight modifications. DPPH radical solution (2.9 ml of 0.1 mM) was mixed with varying concentration (0.1 ml of 1 ‐ 10 mg/ml) of the extracts or gallic acid used as reference and incubated in the dark for 30 min. The control solution consisted of 0.1 ml of 90% v/v methanol in lieu of the samples. The absorbance was read at 517 nm against the blank (90% v/v methanol). Analyses were carried out in triplicates. Percentage inhibition of DPPH radical was calculated as:

$$\begin{array}{cc} \%\;\text{Radical Scavenging Activity}= & \left({\text{Abs}}_{\mathrm{control}}-{\text{Abs}}_{\mathrm{sample}}\right)/\left({\text{Abs}}_{\mathrm{control}}\right)\\ & \times 100 \end{array}$$

Mean effective concentration (EC_50_) was also computed using linear regression analysis.

### 2.7 Determination of total antioxidant capacity

Total antioxidant capacity was carried out using phosphomolybdenum assay (Prieto, Pineda, & Aguilar, 1999). CN‐M and CN‐E samples (0.1 ml of 1 mg/ml) were mixed with 1 ml of the phosphomolybdenum reagent (0.6 M sulfuric acid, 28 mM sodium phosphate, and 4 mM ammonium molybdate). The tube was capped and incubated in a water bath at 95°C for 90 min. After cooling the sample to room temperature, the absorbance of the aqueous solution was measured at 695 nm against blank. The blank solution contained 1 ml of reagent solution and the appropriate volume of the same solvent used to dissolve the extracts. Total antioxidant capacity was expressed as equivalents of the gallic acid used as reference.

### 2.8 Evaluation of alpha‐amylase inhibitory activity

Antidiabetic potentials of extracts from *C. nucifera* were evaluated using in vitro α‐amylase inhibitory activity assay according to the method described by previous study (Oboh et al., 2012). Varying concentration (0.1 ml of 10–100 μg/ml) of CN‐E and CN‐M were incubated with 500 μl of 20 mM phosphate buffer (pH, 6.9, with 6 mM NaCl) containing porcine pancreatic α‐amylase (0.5 mg/ml) at 25°C for 30 min, respectively. Then, 500 μl of 1% starch solution prepared in phosphate buffer was added to each tube to initiate the reaction and the reaction mixtures were further incubated at 37°C for 10 min. The reaction was terminated by the addition of 0.5 ml of 3,5‐dinitrosalicylic acid (DNS). Thereafter, the mixture was heated in a boiling water bath for 5 min and allowed to cool to room temperature (≤27°C). The resulting mixture was diluted by adding 5 ml of distilled water and absorbance was read at 540 nm against the blank. The control experiment was performed without test sample. Analyses were performed in triplicate and the amylase inhibitory activity was expressed as percentage inhibition using the following formula:

$$\%\;\text{Inhibition of}\;\alpha -\text{amylase}=\left({\text{Abs}}_{\mathrm{control}}-{\text{Abs}}_{\mathrm{sample}}\right)/\left({\text{Abs}}_{\mathrm{control}}\right)\times 100$$

### 2.9 Evaluation of lipid peroxidation inhibitory activity

The potential of CN‐M extract and its CN‐E fraction to prevent end products of lipid peroxidation in compromised pancreatic tissues were evaluated. Sodium nitroprusside (SNP) induced lipid peroxidation bioassay was employed (Ohkawa, Ohishi, & Yagi, 1979). Pancreatic homogenate (0.5 ml) was mixed with a reaction mixture containing the buffer (1.6 ml of 0.1 M pH 7.4 Tris‐HCl) and 0.1 ml of varying concentration (1–100 mg/ml) of test samples. The pro‐oxidant (0.1 ml of 0.1 mM SNP) was added to the mixture and incubated at 37°C for 2 h. The reaction was stopped by addition of sodium dodeacyl sulfate (0.5 ml of 8.1% SDS), followed by the addition of trichloroacetic acid (1 ml of 10% TCA) and thiobarbituric acid (1 ml of 0.8% TBA) to the reaction mixture. This mixture was incubated for color development at 100°C for 30 min. The control solution consisted of phosphate buffer in lieu of the samples. The TBA reactive species produced were measured at 532 nm and expressed as malondialdehyde (MDA) in units/mg protein.

$$\begin{array}{cc} \text{MDA}= & \left(\text{absorbance}\;\times \;\text{volume of mixture}\right)/(E_{532}\times \\ & \;\;\text{volume of sample}\;\times \;\text{mg protein}) \end{array}$$

where: E_532_ is the molar absorptivity at 532 nm = 1.56 × 10^5^. Analyses were performed in triplicates and capacity of the extracts to inhibit lipid peroxidation was expressed as

$$\%\;\text{Inhibition}=\left({\text{MDA}}_{\mathrm{control}}-{\text{MDA}}_{\mathrm{sample}}\right)\times 100/\left({\text{MDA}}_{\mathrm{control}}\right)$$

### 2.10 In vivo reproducibility study

The albino rats were fasted 18 hours and their fasting blood glucose (FBG) was determined using the glucometer (Accucheck Compact Plus Glucose Meter, Roche Diagnostic, Mannheim, Germany). A single intraperitoneal injection of 150 mg/kg body weight of alloxan monohydrate in physiological saline was employed to induce hyperglycemia. The animals were monitored for 5 days and those with blood glucose above 250 mg/dl were selected randomly into four groups of six rats each and treated intraperitoneally once a day for 4 days as described hereunder:

- CN‐M: alloxan‐induced rats administered 50 mg/kg b.wt of *C. nucifera* methanolic extracts.
- CN‐E: alloxan‐induced rats administered 50 mg/kg b.wt of *C. nucifera* ethyl acetate fractions.
- MET: alloxan‐induced rats administered 50 mg/kg b.wt of metformin used as reference.
- AIC: alloxan‐induced rats administered saline solution to serve as nontreated control.
- NIC: for comparative assessment, non‐induced rats were also administered saline solution.

Rats were anesthetized, sacrificed, and dissected after 4 days of administration. The pancreas was excised, weighed, and homogenized in either phosphate or Tris‐HCl buffer for analyses of activity of α‐amylase and lipid peroxidation, respectively.

### 2.11 Statistical analysis

Statistical analysis of data was done using Graph pad prism version 6.0. Data were expressed as the mean of three replicates ± standard error of mean. Student's *t*‐test was used to compare between control and test experiments, while one analysis of variance followed by Turkey's post hoc test was used for multiple comparison.

## 3 RESULTS

### 3.1 In vitro study

**Figure 2:**
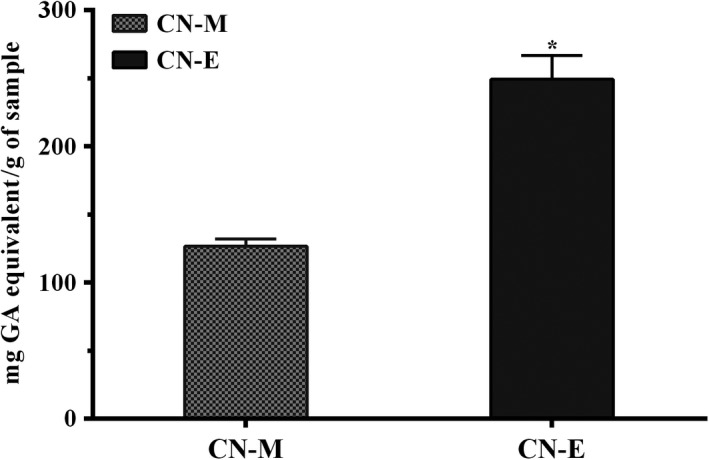
Total pocolyphenolic content of *Cocos nucifera* husk methanolic extract (CN‐M) and its ethyl acetate fraction (CN‐E) *GA: Gallic Acid

**Figure 3:**
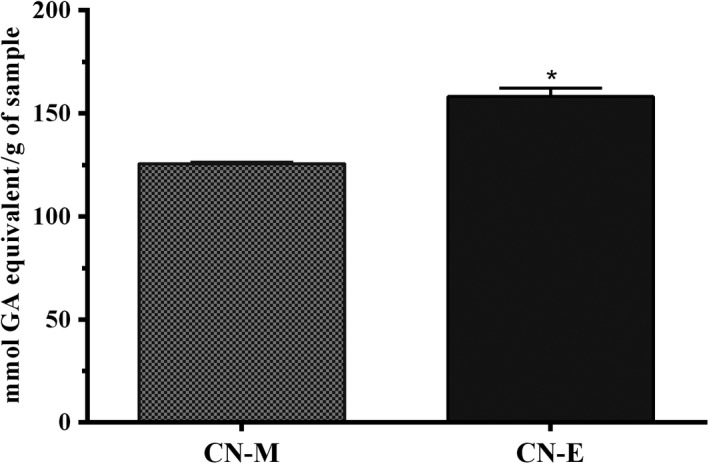
Total antioxidant capacity *Cocos nucifera* methanolic extract (CN‐M) and its ethyl acetate fraction (CN‐E) *GA: Gallic Acid. *Significantly higher at p < 0.05

**Figure 4:**
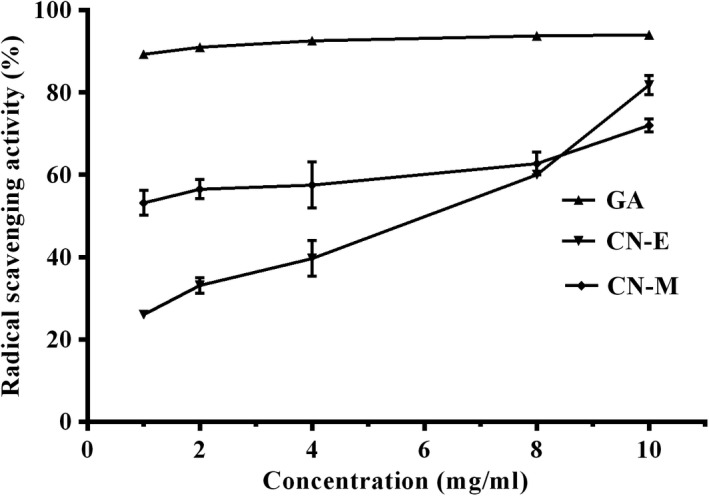
Radical scavenging activity of *Cocos nucifera* methanolic extract (CN‐M) and its Ethyl acetate fraction (CN‐E). *GA: Gallic Acid

Total phenolic content of methanolic extract from *C. nucifera* husk and its ethyl acetate fraction is shown on Figure 2. The phenolic content of the methanolic extract (126.7 ± 5.36 mg gallic acid per gram) was significantly (*p* < 0.05) lower than values determined for its ethyl acetate fraction (249.2 ± 17.51 mg gallic acid per gram). Similarly, the total antioxidant capacity of the ethyl acetate fraction (158.0 ± 4.17 mg gallic acid per gram) was significantly (*p* < 0.05) higher than that of the methanolic extract (125.5 ± 0.78 mg gallic acid per gram; Figure 3). In contrast, lower concentration (≤4 mg/ml) of ethyl acetate fraction showed lower DPPH radical scavenging activity when compared to the methanolic extract, whereas at higher concentration of 10 mg/ml, the activity was higher (Figure 4). Overall, mean effective concentration of the methanolic extract and its ethyl acetate fraction to scavenge 50% of the radical were not significantly (*p* ≥ 0.05) different from each other and the values computed (EC_50_ = ~6 mg/ml) was significantly lower than that of gallic acid (EC_50_ = ~4 mg/ml) used as reference.

**Figure 5:**
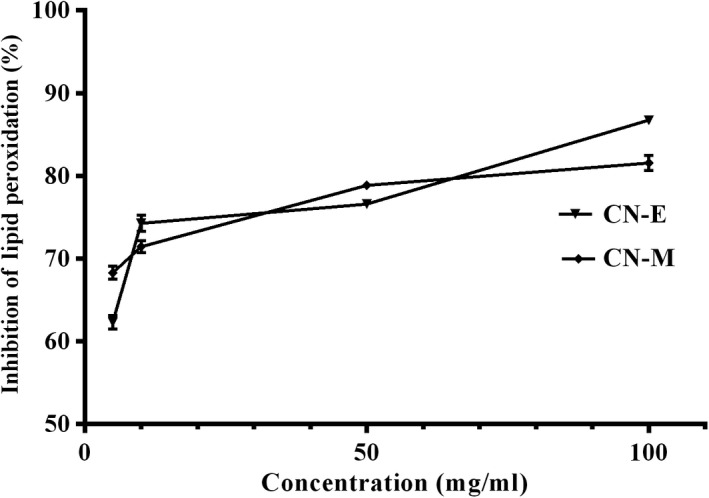
Lipid peroxidation (LP) inhibitory effect of *Cocos nucifera* methanolic extract (CN‐M) and its ethyl acetate fraction (CN‐E)

**Figure 6:**
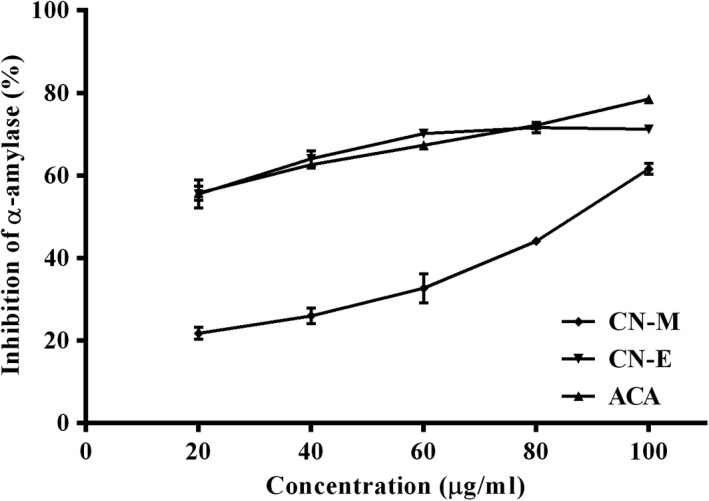
Alpha‐amylase inhibitory activity of *Cocos nucifera* methanolic extract (CN‐M) and its ethyl acetate fraction (CN‐E). *ACA: Acarbose

Figure 5 shows the in vitro lipid peroxidation inhibitory activity of extracts from *C. nucifera* husk. The ability of the extracts to inhibit lipid peroxidation were concentration‐dependent and increased as concentration increases. Similarly, inhibition of alpha‐amylase activity was also concentration dependent (Figure 6). The ethyl acetate fraction (IC_50_ = 53.08 ± 5.35 μg/ml) showed significantly [*F*(2,6) = 49.75; *p* < 0.0002] higher inhibitory effect than the methanolic extract (IC_50_ = 51.70 ± 4.66 μg/ml) and competes favorably with acarbose (IC_50_ = 83.84 ± 2.74 μg/ml) used as reference (Table 1).

**Table 1:** Mean DPPH radical scavenging (EC_50_) and alpha‐amylase inhibitory (IC_50_) concentration of *Cocos nucifera* methanolic extract (CN‐M) and its ethyl acetate fraction (CN‐E)

	DPPH EC_50_ (mg/ml)	α‐amylase IC_50_ (μg/ml)
CN‐M	5.72 ± 0.84^b^	83.84 ± 2.74^b^
CN‐E	5.97 ± 0.37^b^	53.08 ± 5.35^a^
Ref	3.97 ± 0.71^a^	51.70 ± 4.66^a^

*Note*. Ref = Gallic acid and acarbose for DPPH and α‐amylase assays, respectively. *Values with different superscripts^(ab)^ along a column are significantly different from each other at p < 0.0001

### 3.2 In vivo study

Glucose lowering effect of *C. nucifera* methanolic extract and its ethyl acetate fraction in alloxan‐induced hyperglycemic rats is shown on Table 2. Hyperglycemia was significantly (*p* < 0.05) reduced following administration of the extracts and the standard drug (metformin). Normoglycemia was achieved after 5 days of treatment with the ethyl acetate fraction of *C. nucifera* husk, whereas the methanolic extract caused ~75% reduction.

**Table 2:** Glucose lowering effect of *Cocos nucifera* methanolic extract (CN‐M) and its ethyl acetate fraction (CN‐E) in alloxan‐induced hyperglycemic rats

	Before treatment FBG (mg/dL)	After treatment FBG (mg/dL)	Glucose reduction (%)
CN‐M	337.3 ± 4.16^a^	144.7 ± 5.13^b^	74.87
CN‐E	343.5 ± 10.33^a^	89.33 ± 6.66^a^	96.46
MET	354.3 ± 6.34^a^	83.00 ± 1.53^a^	98.91

*Note*. Glucose reduction (%) = [(Before Treatment − After Treatment)/(Before Treatment − Mean Normal Glycemia)]*100; Mean Normal Glycemia = 80 mg/dL; MET = Metformin. *Values with different superscripts (ab) along a column are significantly different from each other at p < 0.0001

**Figure 7:**
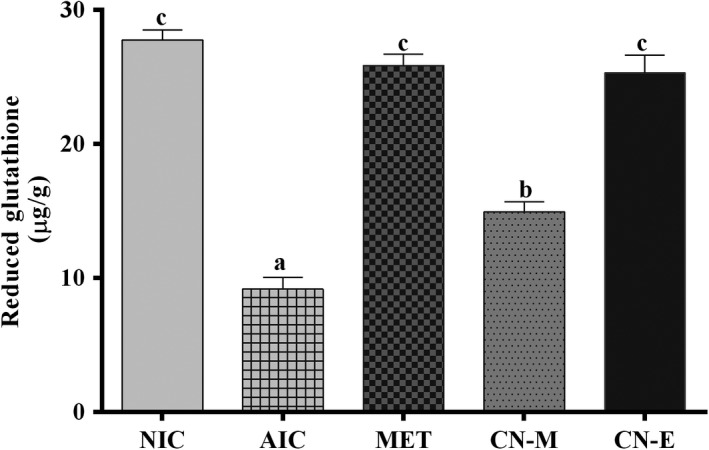
Concentration of reduced glutathione in the pancreas of alloxan‐induced diabetic rats treated with *Cocos nucifera* methanolic extract (CN‐M) and its ethyl acetate fraction (CN‐E). *NIC: Noninduced control; AIC: Alloxan‐induced control; MET: Metformin

**Figure 8:**
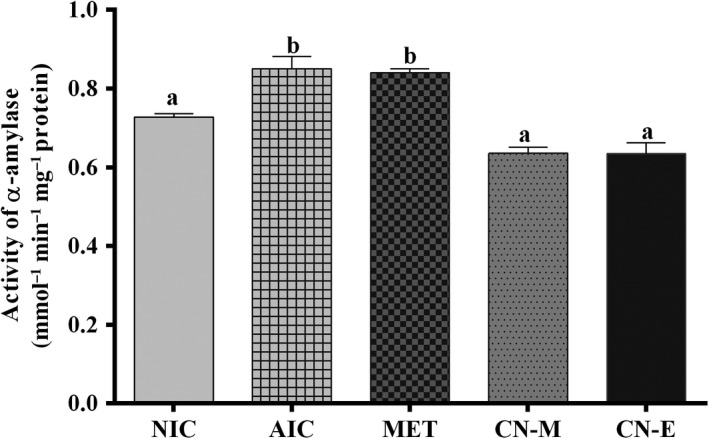
Specific activity of pancreatic alpha‐amylase in alloxan‐induced diabetic rats treated with *Cocos nucifera* methanolic extract (CN‐M) and its ethyl acetate fraction (CN‐E). *NIC: Noninduced control; AIC: Alloxan‐induced control; MET: Metformin

Figure 7 shows pancreatic concentration of reduced glutathione of alloxan‐induced hypergycemic rats treated with extract from *C. nucifera* husk. Administration of alloxan caused a significant reduction in concentration of reduced glutathione (NIC vs AIC; Figure 7). Treatment with either metformin or *C. nucifera* ethyl acetate fraction significantly [*F*(5,15) = 75.72; *p* < 0.0002] increased the concentration of reduced glutathione to values not significantly different from those of non‐induced control group. Conversely, alloxan‐induction significantly increased concentration of malondialdehyde (Figure 8); treatment of the alloxanized rats with the extracts or metformin caused significant [*F*(5,15) = 86.55; *p* < 0.001] reduction in malondialdehyde levels. Concentration of the lipid peroxidation marker in rats following treatment with *C. nucifera* methanolic extracts was not significantly different from those of non‐induced control group.

**Figure 9:**
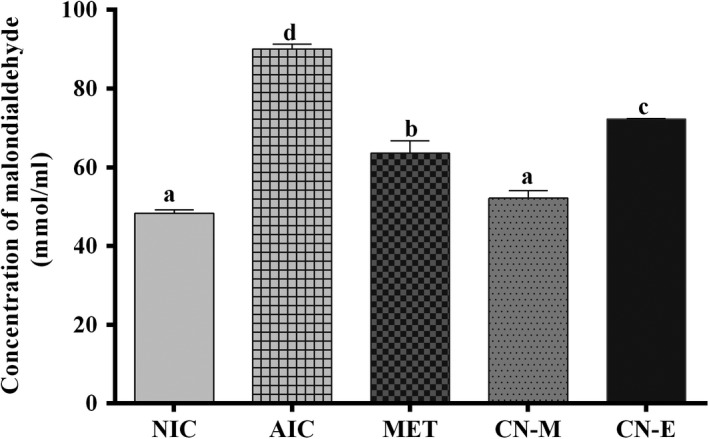
Concentration of malondialdehyde in the pancreas of alloxan‐induced diabetic rats treated with *Cocos nucifera* methanolic extract (CN‐M) and its ethyl acetate fraction (CN‐E) *NIC: Noninduced control; AIC: Alloxan‐induced control; MET: Metformin

Figure 9 shows the specific activity of pancreatic alpha‐amylase in alloxan induced diabetic rats treated with *Cocos nucifera* methanolic extract and its ethyl acetate fraction. Activity of alpha‐amylase was significantly [*F*(5,15) = 25.40; *p* < 0.0001] reduced in alloxan‐induced rats treated with the extracts, whereas activity of the carbohydrate metabolizing enzyme in rats treated with metformin was not significantly different from values determined in the non‐treated group.

## 4 DISCUSSION

Bioactivities of extracts from *C. nucifera* husk have been attributed to its phenolic content (Roopan, 2016). In this study, total phenolic content quantified in *C. nucifera* methanolic extract and its ethyl acetate fraction compared favorably with the amount determined in previous studies (Bezerra dos Santos Oliveira et al., 2013; Khonkarn, Okonogi, Ampasavate, & Anuchapreeda, 2010; Lima et al., 2016). Correlation between phenolic contents of plants and their antioxidant properties has also been established (Bezerra dos Santos Oliveira et al., 2013; Oboh et al., 2017). Phosphomolydenum assay employed to determine the total antioxidant capacity of the plant extracts is a spectroscopic method based on the reduction of Mo (VI) to Mo (V) by the sample analyte and subsequent formation of a green phosphate Mo (V) complex at acidic pH (Prieto et al., 1999). The total antioxidant capacity of *C. nucifera* husk extracts strongly correlated with its total phenolic content. The ethyl acetate fraction with higher phenolic content recorded higher antioxidant capacity. Previous study on DDPH radical scavenging activities of ethanolic extracts from four varieties of *C. nucifera* husk showed that varieties with high total phenolic content showed higher radical scavenging activity, confirming that phenols are likely to be the major contributor to the antioxidant capacity of the husk extract (Bezerra dos Santos Oliveira et al., 2013).

DPPH radical scavenging assay is widely used to test the capacity of plant extracts to act as free radical scavengers as an index of their antioxidant potential (Alam et al., 2013). In this study, extracts from *C. nucifera* husk demonstrated concentration‐dependent capacity to scavenge free radicals. The radical scavenging activities of either the *C. nucifera* methanolic extracts or its ethyl acetate fractions compared favorably with the standard phenol (gallic acid) used as reference. The radical scavenging activity of the extract may also be attributed to its phenolic content with the functional hydroxyl group acting as hydrogen donor, thus interacting with the radical to form a more stable complex. Other studies have also shown the ability of *C. nucifera* husk to scavenge other radicals such as ABTS, nitric oxide, hydrogen peroxide, and superoxide among others as detailed in literature (Lima et al., 2015).

Peroxidation of lipid‐rich biological membranes resulting in generation of free radicals is considered as one of the mode of cellular injury in aerobic organisms subjected to oxidative stress (Oboh & Rocha, 2007). Phenolic compounds have the capacity to protect cells, tissues and organs from oxidizing effects of free radicals produced during energy production or normal metabolic processes in living organisms. Data from this study showed that extracts from *C. nucifera* did not only scavenge free radicals but also inhibits peroxidation of lipids in both in vitro and in vivo models. Study also showed that pretreatment of cisplatin‐induced rats with chloroform extracts of *C. nucifera* husks inhibited lipid peroxidation and enhanced the antioxidant defense system of the experimental animals (Adaramoye, Azeez, & Ola‐Davies, 2016). Formation of lipid peroxidation byproducts such as malondiadehyde has been linked to reduction in concentration of reduced gluthatione in alloxan‐induced rats as evident in this study as well as other studies (Pari & Umamaheswari, 2000). However, treatment of rats with *C. nucifera* extracts increased concentration of the antioxidant biomarker (Figure 7).

Alloxan is a diabetogenic agent that is used to induce hyperglycemia in experimental rats via destruction of pancreatic β‐cells of the islets of langerhans mediated by formation of reactive oxygen species resulting in redox imbalance and lipid peroxidation of pancreatic cells. This may also lead to partial or complete loss of insulin synthesis and thus the development of hyperglycemia (Akolade, Usman, Okereke, & Muhammad, 2014). Intraperitoneal administration of *C. nucifera* methanolic extract and its ethlyacetate fraction caused reduction in hyperglycemia to basal and normoglycemic level, respectively. Previous studies have shown that extracts from various parts of *C. nucifera* plants possess antihyperglycemiac effect (Naskar et al., 2011; Pinto et al., 2015). Also, methanolic extract of the immature *C. nucifera* inflorescence (Renjith, Chikku, & Rajamohan, 2013) as well as the aqueous and ethanolic extracts of the mature mesocarp (Tyagi, Hooda, Hooda, & Malkani, 2015) have been reported to reduce hyperglycemia either via potentiating the action of insulin by modulating the pancreatic secretion of insulin or amelioration of degenerated pancreatic β‐cells.

Another approach for managing diabetes mellitus is to decrease postprandial hyperglycemia by hindering the absorption of glucose through inhibition of the carbohydrate hydrolyzing enzymes, such as alpha‐amylase, in the digestive tract (Kwon, Apostolidis, & Shetty, 2007). From the result, ethyl acetate fraction of *C. nucifera* husk showed considerable inhibitory activity against alpha‐amylase in in vitro model with no significant difference with the reference drug (acarbose; Table 1). Similarly, activity of pancreatic alpha‐amylase in alloxan‐induced hyperglycemic rats was attenuated following treatment with the *C. nucifera* extracts (Figure 8). Metformin used as reference also showed considerable antihyperglycemic effect (~99% glucose reduction) but the drug has no effect on alpha‐amylase activity in vivo*,* probably because the antidiabetic agents acts via other approaches such as decreasing gluconeogenesis and increasing glucose uptake by cells. Gastrointestinal digestion has been shown to enhance antioxidant and alpha‐amylase inhibitory activities of polyphenols (He et al., 2017). More so, phenolic compounds such as catechins and chlorogenic acids that have been quantified in *C. nucifera* husk (Lima et al., 2016) are known not only for their potent antioxidant properties but also for their antihyperglycemic effects (Ong, Hsu, & Tan, 2013; Samarghandian, Azimi‐Nezhad, & Farkhondeh, 2017).

## 5 CONCLUSION

Ethyl acetate fraction of the methanolic extract of *C. nucifera* husk fiber showed a significant inhibitory activity against key enzyme linked to diabetes mellitus (alpha‐amylase) coupled with high antioxidant potential in in vitro models and the efficacies were replicated in vivo. The fraction from the plant can be further explored for the management of diabetes mellitus and its associated complications.

## CONFICT OF INTEREST

The authors declare no conflicts of interest.

## ETHICAL APPROVAL

The research adheres strictly and conforms to the Principles of Laboratory Animal Care (NIH Publication No. 85‐23). The research protocols were requested to and approved by the Department of Biochemistry, University of Ilorin, Ilorin, Nigeria.
